# The Mub1/Ubr2 Ubiquitin Ligase Complex Regulates the Conserved Dsn1 Kinetochore Protein

**DOI:** 10.1371/journal.pgen.1003216

**Published:** 2013-02-07

**Authors:** Bungo Akiyoshi, Christian R. Nelson, Nicole Duggan, Steven Ceto, Jeffrey A. Ranish, Sue Biggins

**Affiliations:** 1 Division of Basic Sciences, Fred Hutchinson Cancer Research Center, Seattle, Washington, United States of America; 2 Molecular and Cellular Biology Program, University of Washington, Seattle, Washington, United States of America; 3 Institute for Systems Biology, Seattle, Washington, United States of America; Duke University, United States of America

## Abstract

The kinetochore is the macromolecular complex that assembles onto centromeric DNA and orchestrates the segregation of duplicated chromosomes. More than 60 components make up the budding yeast kinetochore, including inner kinetochore proteins that bind to centromeric chromatin and outer proteins that directly interact with microtubules. However, little is known about how these components assemble into a functional kinetochore and whether there are quality control mechanisms that monitor kinetochore integrity. We previously developed a method to isolate kinetochore particles via purification of the conserved Dsn1 kinetochore protein. We find that the Mub1/Ubr2 ubiquitin ligase complex associates with kinetochore particles through the CENP-C^Mif2^ protein. Although Mub1/Ubr2 are not stable kinetochore components *in vivo*, they regulate the levels of the conserved outer kinetochore protein Dsn1 via ubiquitylation. Strikingly, a deletion of Mub1/Ubr2 restores the levels and viability of a mutant Dsn1 protein, reminiscent of quality control systems that target aberrant proteins for degradation. Consistent with this, Mub1/Ubr2 help to maintain viability when kinetochores are defective. Together, our data identify a previously unknown regulatory mechanism for the conserved Dsn1 kinetochore protein. We propose that Mub1/Ubr2 are part of a quality control system that monitors kinetochore integrity, thus ensuring genomic stability.

## Author Summary

The flawless execution of cell division is essential to the survival of all organisms. The loss or gain of a single chromosome, the state called aneuploidy, is a hallmark of cancer cells and is the leading cause of spontaneous miscarriages and hereditary birth defects. Segregation is mediated by the kinetochore, the macromolecular complex that assembles on each chromosome and attaches to spindle microtubules to pull chromosomes to opposite poles when cells divide. It is therefore critical to understand how kinetochores are assembled and maintained. Here, we find that the levels of a conserved kinetochore protein are regulated by proteolysis. We propose that cells have quality control systems that ensure kinetochore integrity and thus genome stability.

## Introduction

Accurate chromosome segregation is essential to avoid the aneuploidy that is associated with cancer and birth defects [1]. Segregation is directed by the kinetochore, the macromolecular protein complex that assembles onto the centromeric region of each chromosome and that interacts with spindle microtubules during mitosis and meiosis [2]–[5]. The inner part of the kinetochore binds to centromeric DNA, whereas the outer portion interacts with microtubules. Although greater than 60 kinetochore components have been identified in the budding yeast kinetochore, it remains unclear how the individual proteins assemble onto the centromere to form the macromolecular kinetochore structure [6].

At the base of the kinetochore, the CENP-A centromeric histone H3 variant forms a specific chromatin environment essential for recruiting other kinetochore proteins [7], [8]. Components of the CCAN (constitutive centromere-associated network, e.g. CENP-C) closely associate with CENP-A [9], [10]. These inner kinetochore components are essential for the assembly of the outer kinetochore [11]–[13]. The outer kinetochore possesses microtubule-binding activity mediated through the KNL1 and Ndc80 complexes in the KMN (KNL1, Mis12, Ndc80 complexes) network [14]. Although the Mis12 complex does not directly bind to microtubules, it is important for the assembly of the KMN and may be a keystone to promote outer kinetochore assembly [14]–[20]. Recently, the conserved centromere-binding protein CENP-C^Mif2^ has been shown to link the inner and outer portions of the kinetochore by binding directly to the Mis12 complex [21], [22].

Ubiquitin (Ub)-mediated proteolysis is a widely used cellular system to monitor the quality and quantity of numerous proteins [23], [24]. Ubiquitylation of a substrate requires multiple enzymes. Ubiquitin is first activated by an Ub-activating enzyme (E1), transferred to an Ub-conjugating enzyme (E2), and then conjugated to a substrate via an E3 ligase. Although the E3 enzymes largely dictate substrate specificity, efficient ubiquitylation of target proteins requires additional adaptor or cofactor proteins in some cases (e.g. [25], [26]). Proteolysis of kinetochore proteins appears to be important to ensure genomic stability. In budding yeast and flies, the CENP-A protein that is the foundation of centromeric chromatin and directs kinetochore formation is degraded to ensure its exclusive localization at the centromere [27], [28]. In addition, ubiquitin-mediated degradation of components of the CBF3 and Mis12 complexes regulates kinetochore assembly and function [18], [29], [30].

Ubr2 was identified as an E3 ligase that regulates the level of the transcription factor Rpn4 through ubiquitylation [31]. Ubiquitylation of Rpn4 by Ubr2 requires an additional factor Mub1 [32], so we will refer to the complex as Mub1/Ubr2 throughout this paper. A recent study showed that a ribonucleotide reductase inhibitor, Sml1, is also targeted by Mub1/Ubr2 [33]. Although *mub1*Δ and *ubr2*Δ mutants are viable, *ubr2*Δ mutant cells are sensitive to increased proteasome activity [31] and defective in the degradation of unfolded cytoplasmic protein [34]. It is not clear whether the Mub1/Ubr2 ligase complex targets additional proteins or cellular processes.

Here, we used a recently developed kinetochore purification method to show that the Mub1/Ubr2 E3 ligase complex interacts with kinetochore particles through the conserved CENP-C^Mif2^ protein that links inner and outer kinetochore proteins [15]. We found that Mub1/Ubr2 mediate Dsn1 ubiquitylation and regulate its protein levels, especially when the Dsn1 protein is mutated. In addition, Mub1/Ubr2 become important for viability when kinetochore function is compromised. Taken together, these data suggest that Mub1/Ubr2 regulate Dsn1 levels to ensure kinetochore integrity.

## Results

### Kinetochore particles associate with the E3 ubiquitin ligase Ubr2 through Mub1

**Figure 1:**
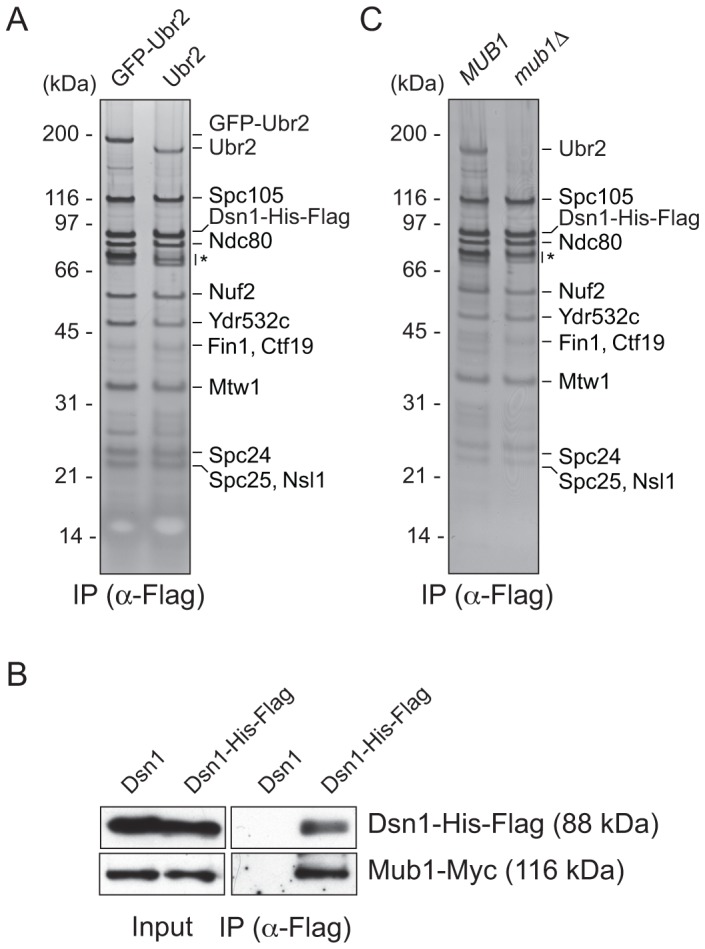
Dsn1 associates with an E3 ubiquitin ligase, Ubr2, via its adaptor, Mub1. (A) Dsn1 associates with Ubr2. Dsn1-His-Flag was purified from cells containing either *pGAL-GFP-UBR2* (SBY8605) or *UBR2* (SBY8253) after 2 hours of growth in galactose and analyzed via silver staining. The prominent band migrating ∼190 kDa is shifted up when GFP-Ubr2 is expressed, confirming that it is Ubr2 (Ubr2: 217 kDa, GFP: 27 kDa). Note that Mub1 runs at the same position as background bands (indicated by asterisk). (B) Co-immunoprecipitation confirms that Dsn1 associates with Mub1. Proteins were purified with anti-Flag antibodies from cells containing Mub1-Myc that express either Dsn1-His-Flag (SBY8550) or untagged Dsn1 (SBY8590) and analyzed by immunoblot. Note that the Dsn1-His-Flag band overlaps with a background signal in the input. (C) The association between Dsn1 and Ubr2 requires Mub1. Dsn1-His-Flag was purified from cells in the presence (SBY8253) or absence (SBY8480) of *MUB1*. The band corresponding to Ubr2 is absent in *mub1Δ* cells. Background bands are indicated by asterisk.

We previously developed a technique to isolate functional kinetochore particles via the purification of the Dsn1 kinetochore protein [15]. Although the particles contain components spanning the inner to outer kinetochore, they lack the centromere-binding complex CBF3 and some other inner kinetochore proteins. These data suggest that the particles are not bound to centromeric DNA after purification. Most of the proteins in the preparations that are visible by silver-stained SDS-PAGE are kinetochore components. However, a ∼190 kDa band that did not correspond to any candidate kinetochore proteins consistently co-purified with the particles (Figure 1A). Notably, mass spectrometry (MS) analysis of purified particles from asynchronously growing cells identified the E3 ubiquitin ligase Ubr2 and its cofactor protein Mub1 that had not previously been implicated in kinetochore function [15]. Because the predicted molecular weight of Ubr2 is 216 kDa, we tested whether the unknown candidate is Ubr2 by purifying kinetochore particles from cells expressing GFP-Ubr2. The band shifted upward when kinetochores were analyzed by silver-stained SDS-PAGE, confirming its identity as Ubr2 (Figure 1A). The Mub1 protein overlaps with non-specifically co-purifying proteins that migrate around 70 kDa (indicated by * in Figure 1A), so we performed an immunoblot to confirm that Mub1 also specifically binds to Dsn1-derived kinetochore particles (Figure 1B). The association between Ubr2 and Dsn1 was abolished in the absence of Mub1 (Figure 1C), suggesting that Mub1 acts as an adaptor protein that recruits Ubr2 onto kinetochore particles. Although this result appears to contrast previous studies that showed that Mub1 is not required for Ubr2 to interact with the Rpn4 protein [26], the interaction with Rpn4 was only tested in the presence of high Ubr2 levels.

### CENP-C recruits Mub1/Ubr2 onto kinetochore particles

**Figure 2:**
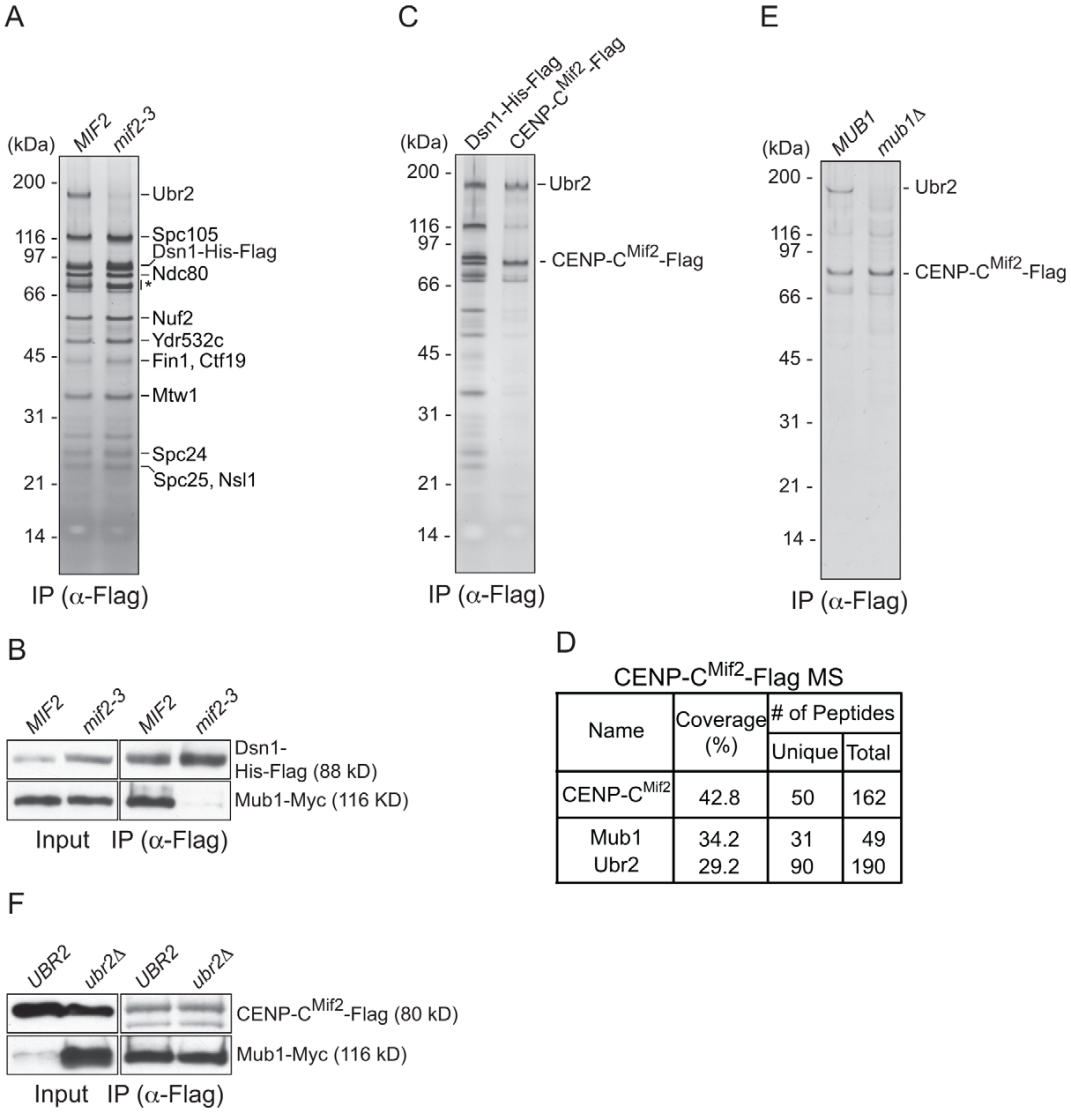
CENP-C recruits Mub1/Ubr2 onto Dsn1-derived kinetochore particles. (A) Dsn1 association with Ubr2 requires CENP-C^Mif2^. Dsn1-His-Flag was immunoprecipitated from cells with either *MIF2* (SBY8253) or *mif2-3* (SBY8405) and analyzed via SDS-PAGE and silver staining. Note that CENP-C^Mif2^ runs at the same position as background bands (indicated by asterisk). (B) Dsn1 association with Mub1 requires CENP-C^Mif2^. Dsn1-His-Flag was immunoprecipitated from cells containing Mub1-Myc and either *MIF2* (SBY8550) or *mif2-3* (SBY8551). (C) CENP-C^Mif2^ associates with Ubr2. Dsn1-His-Flag (SBY8253) and CENP-C^Mif2^-Flag (SBY8519) were immunoprecipitated and analyzed via SDS-PAGE and silver staining. (D) CENP-C^Mif2^-Flag MS summary table. See Table S1 for all proteins identified by MS. (E) CENP-C^Mif2^ association with Ubr2 requires Mub1. CENP-C^Mif2^-Flag was immunoprecipitated from cells in the presence (SBY8519) or absence (SBY8911) of *MUB1* and analyzed via SDS-PAGE and silver staining. (F) The association between CENP-C^Mif2^ and Mub1 does not require Ubr2. CENP-C^Mif2^-Flag was immunoprecipitated from cells containing Mub1-Myc in the presence (SBY8546) or absence (SBY8572) of *UBR2* and analyzed via immunoblot. The Mub1 protein level in the input is higher in *ubr2*Δ due to the lack of Ubr2-dependent proteolysis [26]. Note that the CENP-C^Mif2^-Flag band overlaps with a background signal in the input.

To identify the kinetochore protein that recruits Mub1/Ubr2, we purified kinetochore particles from various kinetochore mutants and assayed for the presence of Ubr2 on a silver-stained gel. Ubr2 associated normally with kinetochore particles purified from outer kinetochore mutants *ndc80-1* (Ndc80 complex), *spc105-15* (KNL1 complex), and *dad1-1*, *ask1-3* (Dam1 complex), as well as inner kinetochore mutants *mcm21Δ*, *okp1-5* (CCAN/COMA complex), and *cse4-323* (CENP-A) (Figure S1 and [15]). In contrast, the association of Mub1/Ubr2 with kinetochore particles was almost completely abolished in the *mif2-3* (CENP-C) mutant (Figure 2A and 2B). To test whether CENP-C^Mif2^ and Mub1/Ubr2 closely associate, we purified the CENP-C^Mif2^-Flag protein. A silver-stained SDS-PAGE gel of purified CENP-C^Mif2^ revealed a band that migrates around 190 kDa in addition to the Mif2-Flag band (Figure 2C). MS analysis of the purified sample showed that Mub1 and Ubr2 are among the most abundant proteins in the sample, suggesting that the 190 kDa band is most likely Ubr2 (Figure 2D and Table S1). Conversely, when Mub1-Flag was purified, several kinetochore proteins including CENP-C^Mif2^ were detected (Figure S2 and Table S2). Our purifications of other kinetochore proteins (e.g. Nuf2, KNL-1^Spc105^, CENP-A^Cse4^, Fin1) did not result in significant enrichment of the Mub1/Ubr2 proteins [15], [35], suggesting that the association of Mub1/Ubr2 depends on a unique feature of CENP-C^Mif2^. The interaction between Mif2 and Ubr2 requires Mub1 (Figure 2E), whereas the interaction between CENP-C^Mif2^ and Mub1 is not dependent on Ubr2 (Figure 2F). These data show that Mub1 requires CENP-C^Mif2^ to recruit the Ubr2 ligase onto kinetochore particles.

### Mub1/Ubr2 mediate Dsn1 ubiquitylation and degradation

**Figure 3:**
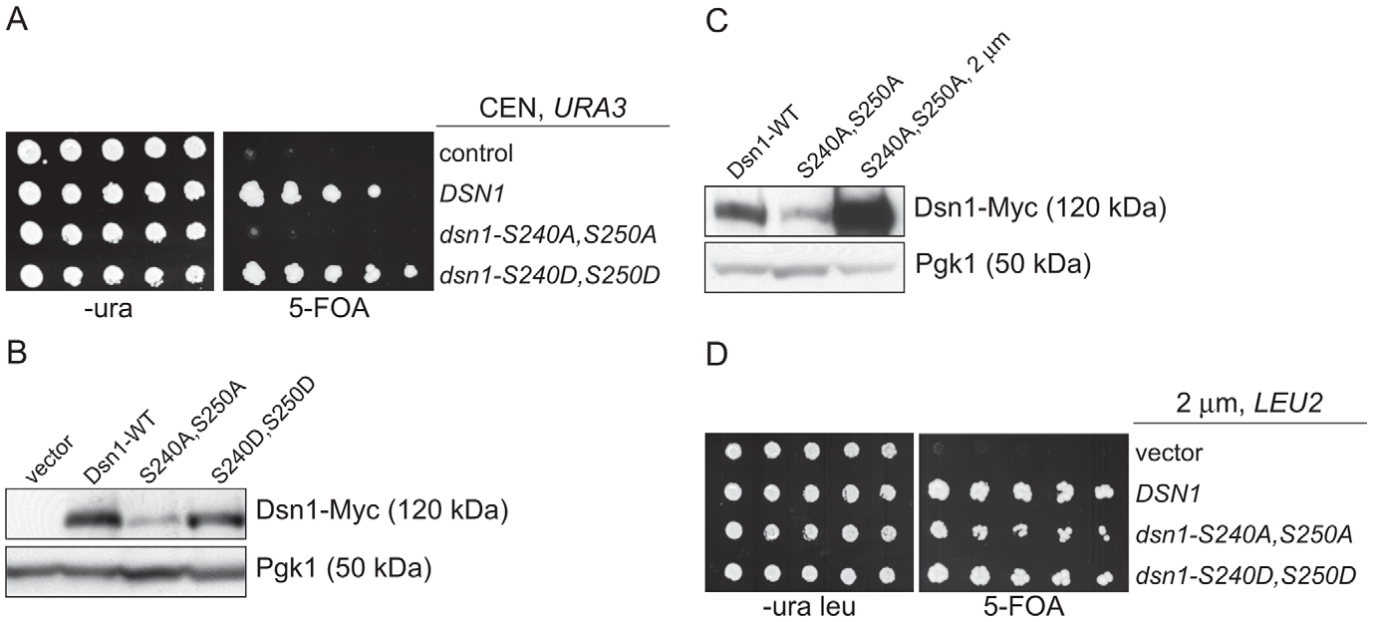
Dsn1-S240A,S250A levels correlate with viability. (A) *Dsn1-S240A,S250A* cells are inviable. Serial dilutions (3-fold) of *dsn1Δ* cells containing *DSN1* on a *URA3*, CEN vector and the indicated integrated point mutants (SBY2318, SBY5948, SBY5949, SBY5950) were plated. Cells that need to maintain the *URA3*, CEN vector for viability are sensitive to 5-FOA. (B) Dsn1-S240A,S250A protein levels are reduced. Whole cell extracts of Dsn1 and the indicated point mutants (SBY2153, SBY7864, SBY7865, SBY7867) were prepared and analyzed via immunoblot. (C) Overexpression of Dsn1-S240A,S250A restores protein levels. Whole cell extracts of the indicated Dsn1 mutants (SBY8766, SBY8521, SBY7373) were prepared and analyzed via immunoblot. (D) Overexpression of *dsn1-S240A,S250A* restores viability. Serial dilutions (3-fold) of *dsn1Δ* strains containing *DSN1* on a *URA3*, CEN vector and a 2 µm *LEU2* plasmid with the indicated point mutants (SBY7368, SBY7362, SBY7363, SBY7364 were plated on –ura leu and 5-FOA plates. We did not detect any obvious defect in cells overexpressing wild-type or mutant Dsn1 proteins.

Our identification of a ubiquitin ligase that associates with kinetochore particles suggests there might be a relevant kinetochore target. Although CENP-C protein is ubiquitylated by the viral immediate-early protein ICP0 upon Herpes Simplex Virus infection [36], we found no evidence that the wild-type budding yeast CENP-C^Mif2^ protein is ubiquitylated *in vivo* despite its close association with the Mub1/Ubr2 ligase complex (data not shown). We therefore considered Dsn1 as a potential target because we isolated an unstable mutant while studying its phosphoregulation (manuscript in preparation). Dsn1 contains two conserved Aurora B kinase consensus sites (S240 and S250) [19], [37], [38] that we mutated to analyze the corresponding phenotypes. When both sites were mutated to alanine to block phosphorylation (*dsn1-S240A,S250A*), the cells were inviable (Figure 3A). In contrast, the phospho-mimic mutant (*dsn1-S240D,S250D*) is viable. To analyze the corresponding protein levels, myc-tagged Dsn1 phospho-mutants were expressed from the endogenous *DSN1* promoter in the presence of wild-type *DSN1* to keep the cells alive. Although the protein levels of wild-type Dsn1 and the phospho-mimic mutant were similar, there were lower levels of the Dsn1-S240A,S250A mutant (Figure 3B). To test whether the inviability of *dsn1-S240A,S250A* is a result of low protein levels, we expressed *dsn1-S240A,S250A* from a high copy plasmid, which led to much higher protein levels (Figure 3C). The overexpressed *dsn1-S240A,S250A* mutant complements *dsn1Δ* (Figure 3D), supporting the idea that the inviability is at least partially due to reduced Dsn1 protein levels.

**Figure 4:**
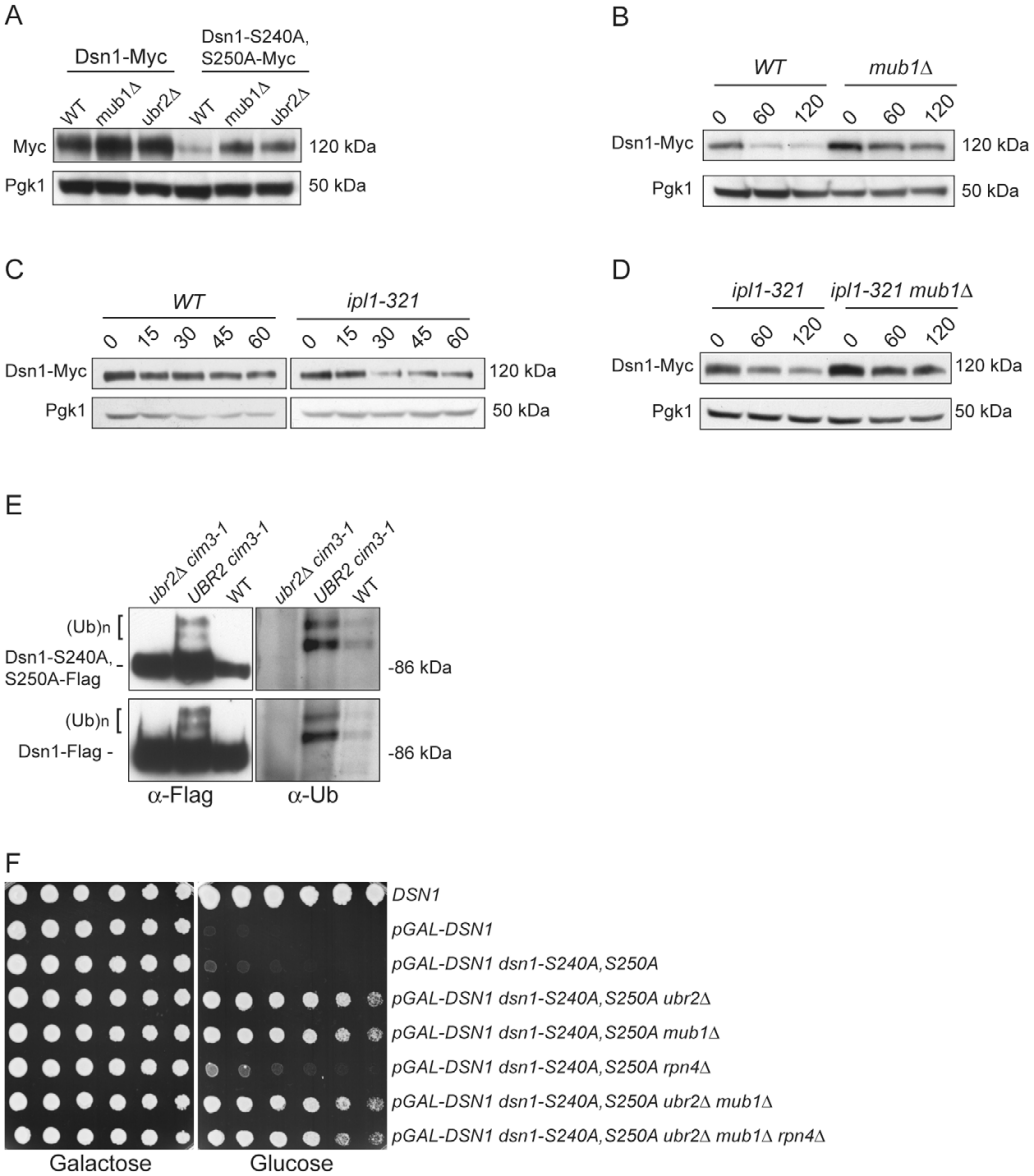
Mub1/Ubr2 mediate Dsn1 ubiquitylation and regulate protein levels. (A) Deleting *MUB1* and *UBR2* restores Dsn1-S240A,S250A protein levels. Whole cell extracts were prepared from WT (SBY8766, SBY8521), *mub1Δ* (SBY10959, SBY8164), and *ubr2Δ* cells (SBY10960, SBY8265). Dsn1-Myc and Dsn1-S240,S250A-Myc levels were monitored by immunoblot. (B) Mub1 regulates Dsn1 stability. WT (SBY8766) and *mub1Δ* (SBY10959) cells containing Dsn1-myc were treated with cycloheximide and analyzed for Dsn1 protein levels at the indicated time points (min). (C) Aurora B regulates Dsn1 stability. WT (SBY8766) and *ipl1-321* (SBY8150) cells containing Dsn1-myc were shifted to 37°C and treated with cycloheximide. Cells were analyzed for Dsn1 protein levels at the indicated time points (min). (D) Mub1/Ubr2 regulate Dsn1 stability in an Aurora B mutant. *Ipl1-321* (SBY8150) and *ipl1-321 mub1Δ* (SBY9428) cells containing Dsn1-myc were shifted to 37°C and treated with cycloheximide. Cells were analyzed for Dsn1 protein levels at the indicated time points (min). (E) Ubr2 ubiquitylates Dsn1-S240A,S250A and wild-type Dsn1. Flag epitope-tagged Dsn1-S240A,S250A or Dsn1-WT was immunoprecipitated from *cim3-1 ubr2Δ* cells (SBY8703, SBY8705), *cim3-1 UBR2* cells (SBY8704, SBY8706) and WT cells (SBY8615, SBY7441). Samples were analyzed via immunoblot with anti-Flag and anti-Ub antibodies. (F) Deleting *MUB1* and *UBR2* restores viability to Dsn1-S240A,S250A cells. Serial dilutions (3-fold) of *pGAL-DSN1* cells containing integrated *dsn1-S240A,S250A* with the indicated deletions (SBY8264, SBY8262, SBY8844, SBY8469, SBY8842, SBY8901) were plated on either glucose or galactose media. A WT strain (SBY3) and a *pGAL-DSN1* strain lacking the integrated point-mutant (SBY7948) were included as controls.

We asked whether Dsn1 is a target of Mub1/Ubr2 by testing whether a deletion of *MUB1* or *UBR2* could stabilize the Dsn1-S240A,S250A mutant protein. Strikingly, Dsn1-S240A,S250A protein levels were restored to near wild-type in both *mub1Δ* and *ubr2Δ* strains (Figure 4A). The levels of WT Dsn1 were also increased in the *mub1Δ* and *ubr2Δ* strains, suggesting that Mub1/Ubr2 mediate the degradation of WT Dsn1 protein. We therefore analyzed Dsn1 stability by adding cycloheximide to repress translation. The Dsn1 protein levels were higher in the *mub1Δ* strain at the start of the experiment and did not decrease as much as in WT cells (Figure 4B), although there is still some degradation in the absence of Mub1/Ubr2. Because Mub1/Ubr2 target the Dsn1-S240A,S250A protein that lacks Aurora B phosphorylation sites, we also analyzed Dsn1 stability in a budding yeast Aurora B mutant, *ipl1-321*. We found that Dsn1 is somewhat less stable in an *ipl1-321* mutant relative to WT cells and that the degradation is at least partially dependent on Mub1/Ubr2 (Figure 4C and 4D). These data are consistent with the lack of phosphorylation on residues 240 and 250 leading to the degradation of Dsn1.

We next tested whether Mub1/Ubr2 directly mediate the ubiquitylation of Dsn1. When purified from a *cim3-1* proteasome mutant, upper forms of WT Dsn1 as well as the Dsn1-S240A,S250A mutant protein that are characteristic of ubiquitylation were apparent on an immunoblot (Figure 4E). Indeed, upper bands specific to a Dsn1 immunoprecipitation were recognized by anti-ubiquitin antibodies and were more abundant in *cim3-1* mutant cells. Importantly, the ubiquitylation of Dsn1 was dependent on the Ubr2 E3 ligase (Figure 4E). Taken together, these data strongly suggest that the Mub1/Ubr2 E3 ligase targets the Dsn1 kinetochore protein for degradation.

Our finding that Mub1/Ubr2 regulate Dsn1 protein levels led us to test whether *mub1Δ* and *ubr2Δ* deletion mutants suppress the lethality of *dsn1-S240A,S250A*. As predicted, there was significant suppression by deletion of either Mub1 or Ubr2 (Figure 4F). The *mub1Δ ubr2Δ* double mutant suppressed to a similar extent as the individual mutants, consistent with Mub1 and Ubr2 functioning in a complex to control Dsn1 protein levels. It was previously found that *mub1Δ* and *ubr2Δ* mutants exhibit increased proteasome activity because the Rpn4 transcription factor is not degraded [26]. As expected, suppression of the *dsn1-S240A,S250A* lethality is not due to increased proteasome activity, because suppression occurred even in the *mub1Δ ubr2Δ rpn4Δ* triple mutant. Taken together, these results show that the *dsn1-S240A,S250A* mutant is lethal because it has reduced protein levels due to Mub1/Ubr2-dependent proteolysis. Our kinetochore particles also interact with another E3 ligase, Psh1 [15], but its deletion does not suppress *dsn1-S240A,S250A* lethality (data not shown), consistent with its specific regulation of CENP-A [39].

### Mub1/Ubr2 are important when kinetochore function is compromised

**Figure 5:**
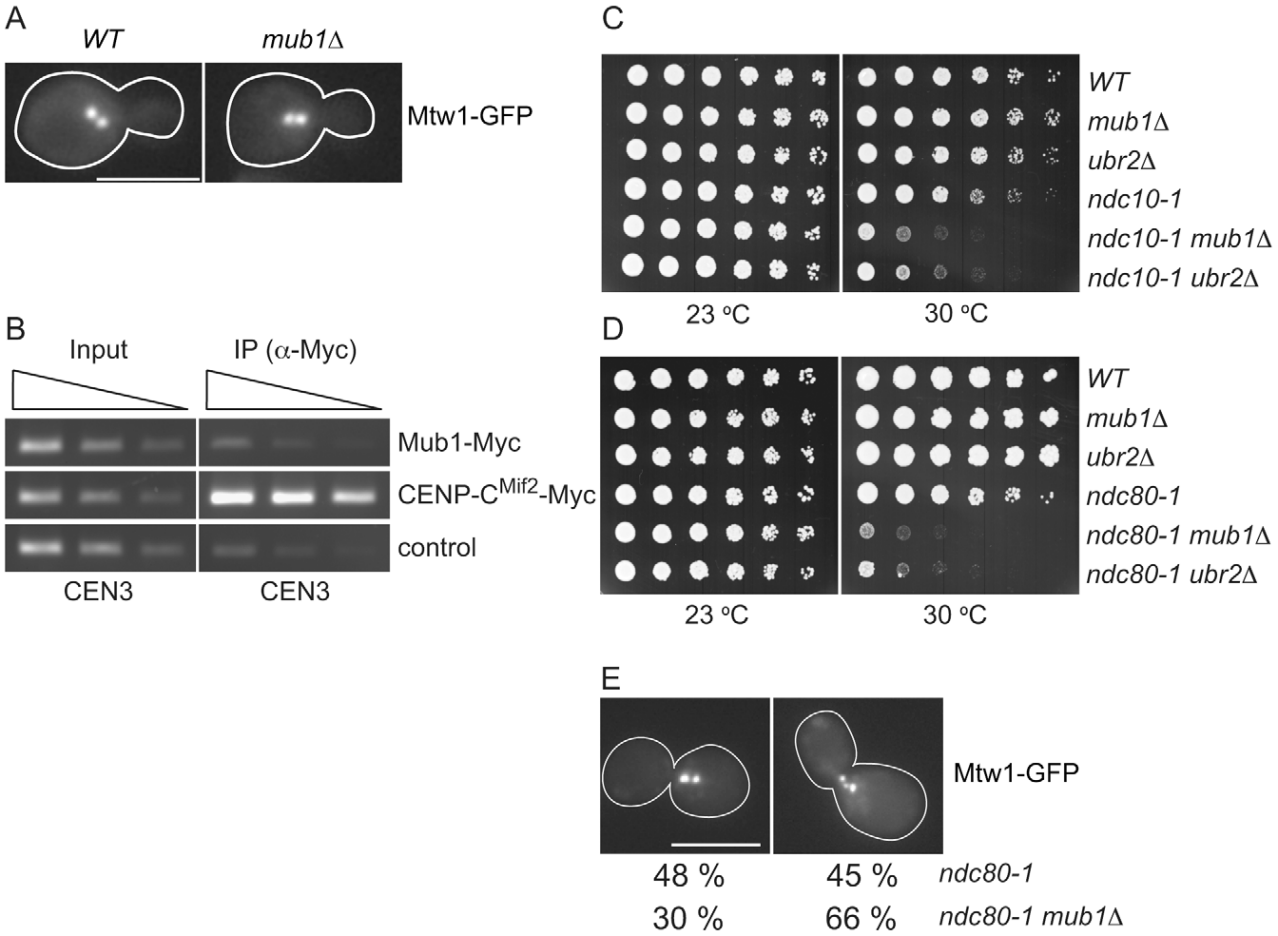
Mub1/Ubr2 cannot be detected at endogenous centromeres but are important for kinetochore function. (A) Mub1 is dispensable for kinetochore biorientation. Mtw1-3GFP was monitored in either *MUB1* (SBY3798) or *mub1Δ* (SBY8026) cells for biorientation defects. A representative cell from each strain is shown. Bar, 5 µm. (B) Mub1 cannot be detected at centromeres. Chromatin immunoprecipitation was carried-out on Mub1-Myc *ubr2Δ* (SBY8572), Mif2-Myc (SBY1566), and untagged control (SBY3) cells using a probe for CEN3. We obtained similar results using Mub1-Myc *UBR2* cells (data not shown). (C, D) *Mub1Δ* and *ubr2Δ* exhibit negative genetic interactions with kinetochore mutants. Serial dilutions (5-fold) of *ndc10-1* (SBY3, SBY7793, SBY7851, SBY164, SBY8613, SBY8773) and *ndc80-1* (SBY3, SBY7793, SBY7851, SBY1117, SBY8436, SBY8432) kinetochore mutants with *mub1*Δ and *ubr2*Δ were plated on YPD and incubated at the indicated temperatures to examine genetic interactions. (E) *Ndc80-1 mub1*Δ double mutants exhibit an increase in declustered kinetochores. *Ndc80-1* (SBY3934) and *ndc80-1 mub1*Δ (SBY8670) cells containing Mtw1-3GFP were released from G1 to 30 degrees and kinetochores were visualized at 180′. The percent of clustered (left panel) vs. unclustered (right panel) was quantified. Bar, 5 µm.

The regulation of Dsn1 by Mub1/Ubr2 prompted us to test whether they have a role in chromosome segregation. Because Mub1 mediates the Mub1/Ubr2 interaction with kinetochores, we analyzed a *mub1Δ* strain. However, chromosome biorientation in metaphase cells (as judged by the characteristic bi-lobed formation of the Mtw1 kinetochore protein [40], Figure 5A) and chromosome segregation (monitored by segregation of a pair of marked sister chromatids [41]) (data not shown) appeared normal in *mub1Δ* mutant cells. Interestingly, we failed to identify Mub1/Ubr2 by MS or immunoblot when we purified centromeric minichromosomes [35], suggesting they may not be core kinetochore components. To determine whether Mub1/Ubr2 localize to endogenous kinetochores, we performed chromatin immunoprecipitation (Figure 5B) and chromosome spread experiments (data not shown). We did not detect an association of Mub1/Ubr2 with endogenous kinetochores, indicating that Mub1/Ubr2 are not structural components of kinetochores. However, they may transiently associate with kinetochores.

Because we did not detect a major defect in segregation in the absence of Mub1/Ubr2, we considered the possibility that Mub1/Ubr2 becomes important when kinetochore function is compromised. We analyzed the *ndc10-1* temperature sensitive mutant that is completely defective in kinetochore assembly [42] as well as the *ndc80-1* mutant that assembles a kinetochore with defects specifically outer kinetochore function [43]. Strikingly, deletion of Mub1/Ubr2 decreased the semi-permissive temperature of these mutants, indicating that their function is important for viability when kinetochores are defective (Figure 5C and 5D). To determine whether the growth defect in *ndc80-1 mub1Δ* strains is due to defective kinetochore function, we monitored kinetochore clustering in the double mutant. Although kinetochores in wild-type yeast cells cluster into two distinct foci at metaphase [40], [44], [45], cells with defects in microtubule-kinetochore attachments exhibit three or more declustered foci [46]. We visualized kinetochores using GFP epitope-tagged Mtw1 kinetochore protein in *ndc80-1* and *mub1Δ ndc80-1* mutant strains. Cells were released from G1 into the cell cycle at 30 degrees and monitored for kinetochore foci after 140 min (Figure 5E). When the number of kinetochore foci were quantified, 45% of the *ndc80* mutant cells exhibited declustered kinetochores (greater than 3 foci) compared to 66% in the *ndc80 mub1* cells. These data are consistent with greater than 75% of the cells being arrested in metaphase with high Pds1 levels due to spindle checkpoint activation. Taken together, these data suggest that kinetochore integrity is further compromised by the lack of Mub1 in the *ndc80-1* mutant cells.

## Discussion

Here, we show that the Mub1/Ubr2 E3 ligase complex regulates the levels of the conserved Dsn1 kinetochore protein via ubiquitin-mediated proteolysis. Mub1/Ubr2 have previously been implicated in proteasome function through the regulation of Rpn4 [26], [31], as well as regulation of dNTP levels through Sml1 degradation [33]. Our data suggest that Mub1/Ubr2 also regulate kinetochore function through proteolysis of the Dsn1 protein. Although Mub1/Ubr2 requires the Rad6 E2 protein to target Rpn4 and Sml1 [26], [33], a *rad6Δ* does not suppress the lethality of *dsn1-S240A,S250A* (data not shown), suggesting that Mub1/Ubr2 utilizes a different or additional E2 to target the Dsn1 kinetochore protein. Identification of the E2 that facilitates the ubiquitylation of Dsn1 should shed additional light on the mechanism by which Mub1/Ubr2 target Dsn1 for degradation.

Our data suggest that CENP-C^Mif2^ is the receptor for Mub1/Ubr2 on kinetochore particles. Kinetochores purified from *mif2* mutants lacked Mub1/Ubr2, and the purification of CENP-C^Mif2^, but not other kinetochore proteins (e.g. Nuf2, KNL-1^Spc105^, CENP-A^Cse4^, Fin1), resulted in a signification enrichment of the Mub1/Ubr2 proteins [15], [35]. Therefore, a unique feature of CENP-C^Mif2^ or a CENP-C^Mif2^-binding protein is important for the interaction of Mub1/Ubr2 with kinetochore particles. However, despite the close association of Mub1/Ubr2 with CENP-C^Mif2^, we did not detect ubiquitylation of CENP-C^Mif2^ or a CENP-C^Mif2^ mutant allele ([47] and data not shown). Because we could not detect a stable association of Mub1/Ubr2 with endogenous kinetochores, Mub1/Ubr2 likely bind to kinetochore particles during the purification process. This raises the possibility that Mub1/Ubr2 only transiently associate with kinetochores to regulate Dsn1, and/or that they regulate a pool of Dsn1 that is not associated with kinetochores.

Although Mub1/Ubr2 are not essential during mitotic growth, a deletion of *mub1* or *ubr2* can restore both the protein levels and inviability of the *dsn1-S240A,S250A* mutant. These data indicate that Mub1/Ubr2 have a physiological role in regulating kinetochore function *in vivo* despite their lack of enrichment at endogenous kinetochores. In addition, deletions in Mub1/Ubr2 exhibited negative genetic interactions with the *ndc80-1* and *ndc10-1* mutants. In the *ndc80* mutant cells, kinetochore-microtubule attachments and biorientation defects were exacerbated by the lack of Mub1. This is reminiscent of the spindle checkpoint proteins that are also not essential in budding yeast but become critical to ensure genomic stability when kinetochore-microtubule interactions are compromised [48], [49]. Mub1 and Ubr2 mutants display stronger meiotic phenotypes [50], and we note that the monopolin complex that joins sister kinetochores during meiosis I to ensure that sister chromatids segregate to the same pole associates with kinetochores via its interaction with CENP-C and Dsn1 [51]. It will be interesting to determine if the Mub1/Ubr2 complex regulates monopolin binding to kinetochores in meiosis. In addition, although monopolin components are sequestered in the nucleolus during mitosis, Mub1/Ubr2 may provide a back up system to prevent them from linking sister kinetochores during mitosis. An important future goal will be to understand the cellular location of the Mub1/Ubr2 ubiquitylation of Dsn1.

We speculate that Mub1/Ubr2 monitor kinetochore integrity and only become important for mitosis when kinetochores are defective. We were not able to detect a significant Mub1/Ubr2-dependent change in Dsn1 stability in *ndc10-1* and *ndc80-1* kinetochore mutants (data not shown), possibly because there is only a small pool of Dsn1 that is regulated by Mub1/Ubr2. In mammalian cells, Dsn1 is regulated by the SCF-Skp1 ubiquitin ligase [18], so it will be important to test whether Skp1-mediated degradation also regulates Dsn1 in budding yeast. We also detected ubiquitylation and regulation of WT Dsn1 protein levels by Mub1/Ubr2, although the effect was mild. We presume that WT cells would not have a need for robust regulation of Dsn1 by Mub1/Ubr2. A deletion of Mub1/Ubr2 led to a strong increase in the levels of the Dsn1-S240A,S250A mutant lacking Aurora B phosphorylation sites. Degradation of mutant alleles is a hallmark of quality control systems. Some temperature sensitive mutants, although possessing normal or near normal activity, are recognized as aberrant and destroyed by quality control mechanisms [52]–[56]. Inhibition of the degradation suppresses the temperature sensitivity, similar to the suppression of *dsn1-S240A,S250A* inviability by deletion of Mub1/Ubr2. One possibility is that kinetochore quality control may be needed to ensure that defective kinetochores do not assemble, or that they are turned over in an attempt to assemble functional kinetochores. For example, *ndc80-1* and *ndc10-1* mutant cells could accumulate aberrant kinetochores in *mub1Δ* and *ubr2Δ* mutants, enhancing their temperature sensitivity. It was recently shown that CENP-C^Mif2^ mediates the interaction between the centromere and outer kinetochore via its interaction with the Mis12 complex that contains Dsn1 [21], [22]. One possibility is that Mub1/Ubr2 binding to CENP-C^Mif2^ regulates kinetochore assembly by preventing stable association of the outer kinetochore via Dsn1-mediated degradation. Consistent with this model, Dsn1 mutants lacking Aurora B phosphorylation sites in other organisms exhibit defects in kinetochore assembly [57], [58]. We speculate that this mechanism would help to avoid the formation of ectopic kinetochores outside of the centromere, as well as play a key role in preventing aberrant kinetochores from stably assembling at centromeres.

There are numerous examples in mammalian studies where mutant kinetochore proteins show decreased protein levels, so it will be important to determine whether a similar quality control system operates in other eukaryotes [16], [59]. Mub1 has a conserved MYND domain that is implicated in mediating protein-protein interactions [60], and MYND-containing proteins are implicated in protein ubiquitylation in human cells [61], [62]. Importantly, many of the MYND-containing proteins are associated with cancer and other diseases (e.g. ZMYND10, ZMYND11/BS69, ETO (eight-twenty-one)/MTG (myeloid translocation gene) family members, DEAF1, and Suppressin (reviewed in [63]). In addition, the stability of Dsn1 orthologs appears to be carefully regulated by chaperones and proteolysis [16], [18], so it will be critical to understand how these MYND proteins function and whether they also regulate kinetochores in mammalian cells. Further studies of potential kinetochore quality control mechanisms could shed light on the process of kinetochore assembly as well as the maintenance of genomic stability in all organisms.

## Materials and Methods

### Yeast strains, plasmids, and microbial techniques

Media and genetic and microbial techniques were essentially as described [64]. Yeast strains and plasmids used in this study are listed in Tables S3 and S4. The *mif2-3* [65], *cim3-1* [66], *ndc10-1* [42], *ndc80-1* [43], , *okp1-5* [46], [68], *dad1-1* [69], *ask1-3* [70], and *cse4-323* [71] alleles were crossed to make strains for this study. Deletions, as well as 3Flag, 13Myc, GFP epitope tags were made using a PCR-based integration system and confirmed by PCR [72]–[74]. Specific primer sequences are listed in Table S5.

### Plasmid construction

pSB1097 (*DSN1*, *HIS3*, integrating vector) was constructed by subcloning *DSN1* with its endogenous promoter from pSB624 [75] into pRS303 (*HIS3*, integrating vector) [76] using *Eco*RI and *Xho*I. pSB1322 (*DSN1-12myc*, *LEU2*, 2 micron plasmid) was constructed by subcloning *DSN1-12myc* with the endogenous *DSN1* promoter of pSB1110 (*DSN1-12myc*, *URA3*, integrating vector) [15], [77] into pRS425 (*LEU2*, 2 micron plasmid) using *Xho*I and *Sac*II. Phospho-mutants were made by Quickchange site-directed mutagenesis (Stratagene).

### Protein and immunological techniques

Immunoprecipitation was performed using BH/0.15 (25 mM HEPES pH 8.0, 2 mM MgCl_2_, 0.1 mM EDTA pH 8.0, 0.5 mM EGTA pH 8.0, 0.1% NP-40, 150 mM KCl, 15% glycerol) containing protease inhibitors, phosphatase inhibitors as described [35]. Immunoblotting was performed as described [78]. Anti-Flag antibodies (M2, Sigma-Aldrich) were used at 1∶3,000, anti-Myc antibodies at 1∶10,000 (9E10, Covance), and anti-Pgk1 (Invitrogen) at 1∶10,000. Anti-Mif2 (OD2, 1∶6,000) antibodies were kind gifts from Arshad Desai [35]. To detect ubiquitylation of Dsn1, 10 mM N-ethyl maleimide was added to the buffer throughout purifications. In addition, the nitrocellulose membrane was autoclaved after the transfer to increase sensitivity [79], and anti-Ubiquitin antibodies (Zymed) were used at 1∶500. Silver-staining was performed on 4–12% NuPAGE Novex Bis-Tris gels (Invitrogen) using a SilverQuest silver-staining kit according to instructions (Invitrogen). Chromatin immunoprecipitation was performed using anti-c-Myc antibodies (A14, Santa Cruz Biotechnology) as described [75]. To identify co-purifying proteins, associated proteins were eluted with detergent and analyzed by MS with LTQ-Orbitrap as described [35]. Stability experiments were performed by adding 50 microgram/ml cycloheximide.

### Microscopy

Analysis of Mtw1-3GFP and fluorescently marked sister chromatids were performed as described using a Nikon microscope [46]. Data was collected with two second exposures using Metamorph software. At least 300 cells were analyzed for all reported experiments.

## Supporting Information

Figure S1 Ubr2 associates with Dsn1-derived kinetochore particles purified from various mutants. Dsn1-His-Flag was immunoprecipitated from indicated kinetochore mutants and analyzed via SDS-PAGE and silver staining. The following strains were used: SBY8253 (WT), SBY8381 (*spc105-15*), SBY8368 (*mcm21*Δ), SBY8944 (*dad1-1*), SBY8366 (*ask1-3*), SBY8552 (*okp1-5*), SBY8554 (*cse4-323*). Background bands are indicated by asterisk.
(EPS)

Figure S2 Mub1 associates with kinetochore proteins *in vivo*. Mub1-Flag was purified from asynchronously growing cells (SBY8565). (A) The sample was analyzed by silver-stained SDS-PAGE (left). Note that little Mub1-Flag was eluted off the beads under the mild elution condition compatible for MS. (B) Co-purifying proteins were identified by LC-MS/MS (right). See Table S2 for all proteins identified by mass spectrometry. (C) Co-immunoprecipitation experiments confirmed that Mub1 specifically associates with Mif2. An untagged strain (SBY3) was used as a control.
(EPS)

Table S1 Mif2-Flag MS list.
(XLS)

Table S2 Mub1-Flag MS list.
(XLS)

Table S3 Yeast strains used in this study. All strains are isogenic with the W303 background. Plasmids are indicated in brackets.
(DOCX)

Table S4 Plasmids used in this study.
(DOCX)

Table S5 Oligonucleotides used in this study.
(DOCX)
