# Supplementary material for: The Mub1/Ubr2 Ubiquitin Ligase Complex Regulates the Conserved Dsn1 Kinetochore Protein
Source: PLoS Genet. 2013 Feb 7;9(2):e1003216. doi: 10.1371/journal.pgen.1003216 (PMC3567142; doi:10.1371/journal.pgen.1003216)
Supplement: Table S3 — Yeast strains used in this study. All strains are isogenic with the W303 background. Plasmids are indicated in brackets. (DOCX) [file pgen.1003216.s005.docx]

**Table S3**. Yeast strains used in this study. All strains are isogenic with the W303 background. Plasmids are indicated in brackets.

| Strain | Genotype |
| --- | --- |
| SBY3 | *MAT****a*** *ura3-1 leu2,3-112 his3-11 trp1-1 ade2-1 LYS2 can1-100 bar1∆* |
| SBY164 | *MAT****a*** *ura3-1 leu2,3-112 his3-11 trp1-1 ade2-1 LYS2 can1-100 ndc10-1* |
| SBY1117 | *MAT****a*** *ura3-1 leu2,3-112 his3-11 trp1-1 ade2-1 LYS2 can1-100 ndc80-1* |
| SBY1566 | *MAT****a*** *ura3-1 leu2,3-112 his3-11 trp1-1 ade2-1 LYS2 can1-100 bar1∆ MIF2-13myc::KAN* |
| SBY2153 | *MAT****a*** *ura3-1 leu2,3-112 his3-11 trp1-1 ade2-1 LYS2 can1-100 bar1∆ DSN1-3HA::HIS3* |
| SBY2318 | *MAT****a*** *ura3-1 leu2,3-112 his3-11 trp1-1 ade2-1 LYS2 can1-100 bar1∆ dsn1∆KAN [DSN1, CEN, URA3 (pSB624)]* |
| SBY3798 | *MAT****a*** *ura3-1 leu2,3-112 his3-11 trp1-1 ade2-1 LYS2 can1-100 bar1∆ MTW1-3GFP::HIS3* |
| SBY3934 | *MAT****a*** *ura3-1 leu2,3-112 his3-11 trp1-1 ade2-1 LYS2 can1-100 bar1∆ MTW1-3GFP::HIS3 ndc80-1* |
| SBY5948 | *MAT****a*** *ura3-1 leu2,3-112 his3-11::DSN1::HIS3 trp1-1 ade2-1 LYS2 can1-100 bar1*∆ *dsn1∆KAN [DSN1, CEN, URA3 (pSB624)]* |
| SBY5949 | *MAT****a*** *ura3-1 leu2,3-112 his3-11::dsn1-S240A,S250A::HIS3 trp1-1 ade2-1 LYS2 can1-100 bar1∆ dsn1∆KAN [DSN1, CEN, URA3 (pSB624)]* |
| SBY5950 | *MAT****a*** *ura3-1 leu2,3-112 his3-11::dsn1-S240D,S250D::HIS3 trp1-1 ade2-1 LYS2 can1-100 bar1∆ dsn1∆KAN [DSN1, CEN, URA3 (pSB624)]* |
| SBY7362  SBY7363  SBY7364  SBY7368  SBY7373 | *MAT****a*** *ura3-1 leu2,3-112 his3-11 trp1-1 ade2-1 LYS2 can1-100 bar1∆ dsn1∆KAN [DSN1, CEN, URA3 (pSB624)] [DSN1-12myc, 2μm, LEU2 (pSB1322)]*  *MAT****a*** *ura3-1 leu2,3-112 his3-11 trp1-1 ade2-1 LYS2 can1-100 bar1∆ dsn1∆KAN [DSN1, CEN, URA3 (pSB624)] [dsn1-S240A,S250A-12myc, 2μm, LEU2 (pSB1323)]*  *MAT****a*** *ura3-1 leu2,3-112 his3-11 trp1-1 ade2-1 LYS2 can1-100 bar1∆ dsn1∆KAN [DSN1, CEN, URA3 (pSB624)] [dsn1-S240D,S250D-12myc, 2μm, LEU2 (pSB1324)]*  *MAT****a*** *ura3-1 leu2,3-112 his3-11 trp1-1 ade2-1 LYS2 can1-100 bar1∆ dsn1∆KAN [DSN1, CEN, URA3 (pSB624)] [2μm, LEU2 (pRS425)]*  *MAT****a*** *ura3-1 leu2,3-112 his3-11 trp1-1 ade2-1 LYS2 can1-100 bar1∆ dsn1∆KAN [dsn1-S240A,S250A-12myc, 2μm, LEU2 (pSB1323)]* |
| SBY7441 | *MAT****a*** *ura3-1 leu2,3-112 his3-11 trp1-1 ade2-1 lys2∆ can1-100 bar1∆ DSN1-3FLAG::KAN* |
| SBY7793 | *MAT****a*** *ura3-1 leu2,3-112 his3-11 trp1-1 ade2-1 LYS2 can1-100 bar1∆ mub1∆HIS3* |
| SBY7851 | *MAT****a*** *ura3-1 leu2,3-112 his3-11 trp1-1 ade2-1 LYS2 can1-100 bar1∆ ubr2∆HIS3* |
| SBY7864 | *MAT****a*** *ura3-1::DSN1-12myc::URA3 leu2,3-112 his3-11 trp1-1 ade2-1 LYS2 can1-100 bar1∆ DSN1-3HA::HIS3* |
| SBY7865 | *MAT****a*** *ura3-1::dsn1-S240A,S250A-12myc::URA3 leu2,3-112 his3-11 trp1-1 ade2-1 LYS2 can1-100 bar1∆ DSN1-3HA::HIS3* |
| SBY7867 | *MAT****a*** *ura3-1::dsn1-S240D,S250D-12myc::URA3 leu2,3-112 his3-11 trp1-1 ade2-1 LYS2 can1-100 bar1∆ DSN1-3HA::HIS3* |
| SBY7948 | *MAT****a*** *ura3-1 leu2,3-112 his3-11 trp1-1 ade2-1 LYS2 can1-100 bar1∆ pGAL-3HA-DSN1::KAN* |
| SBY8026 | *MAT****a*** *ura3-1 leu2,3-112 his3-11 trp1-1 ade2-1 LYS2 can1-100 bar1∆ MTW1-3GFP::HIS3 mub1∆HIS3* |
| SBY8150 | *MAT****a*** *ura3-1 leu2,3-112 his3-11 trp1-1 ade2-1 LYS2 can1-100 bar1∆*  *DSN1-13MYC::KAN ipl1-321* |
| SBY8164 | *MAT****a*** *ura3-1::dsn1-S240A,S250A-12myc::URA3 leu2,3-112 his3-11 trp**1-1 ade2-1 lys2∆ can1-100 bar1∆ mub1∆HIS3* |
| SBY8253 | *MAT****a*** *ura3-1 leu2,3-112 his3-11 trp1-1 ade2-1 LYS2 can1-100 bar1∆ DSN1-HIS-FLAG::URA3* |
| SBY8262 | *MAT****a*** *ura3-1::dsn1-S240A,S250A-12myc::URA3 leu2,3-112 his3-11 trp1-1 ade2-1 lys2∆ can1-100 bar1∆ pGAL-3HA-DSN1::KAN ubr2∆HIS3* |
| SBY8264 | *MAT****a*** *ura3-1::dsn1-S240A,S250A-12myc::URA3 leu2,3-112 his3-11 trp1-1 ade2-1 LYS2 can1-100 bar1∆ pGAL-3HA-DSN1::KAN* |
| SBY8265 | *MAT****a*** *ura3-1::dsn1-S240A,S250A-12myc::URA3 leu2,3-112 his3-11 trp1-1 ade2-1 lys2∆ can1-100 bar1∆ ubr2∆HIS3* |
| SBY8366 | *MAT****a*** *ura3-1 leu2,3-112 his3-11 trp1-1 ade2-1 LYS2 can1-100 bar1∆ DSN1-HIS-FLAG::URA3 ask1-3* |
| SBY8368 | *MAT****a*** *ura3-1 leu2,3-112 his3-11 trp1-1 ade2-1 LYS2 can1-100 bar1∆ DSN1-HIS-FLAG::URA3 mcm21∆* |
| SBY8381 | *MAT****a*** *ura3-1 leu2,3-112 his3-11 trp1-1 ade2-1 LYS2 can1-100 bar1∆ DSN1-HIS-FLAG::URA3 spc105-15* |
| SBY8405 | *MAT****a*** *ura3-1 leu2,3-112 his3-11 trp1-1 ade2-1 LYS2 can1-100 bar1∆ DSN1-HIS-FLAG::URA3 mif2-3* |
| SBY8432 | *MAT****a*** *ura3-1 leu2,3-112 his3-11 trp1-1 ade2-1 LYS2 can1-100 bar1∆ ubr2∆HIS3 ndc80-1* |
| SBY8436 | *MAT****a*** *ura3-1 leu2,3-112 his3-11 trp1-1 ade2-1* *lys2∆ can1-100 bar1∆ mub1∆HIS3 ndc80-1* |
| SBY8469 | *MAT****a*** *ura3-1::dsn1-S240A,S250A-12myc::URA3 leu2,3-112 his3-11 trp1-1 ade2-1 lys2∆ can1-100 bar1∆ pGAL-3HA-DSN1::KAN rpn4∆HIS3* |
| SBY8480 | *MAT****a*** *ura3-1 leu2,3-112 his3-11 trp1-1 ade2-1 lys2∆ can1-100 bar1∆ DSN1-HIS-FLAG::URA3 mub1∆HIS3* |
| SBY8519 | *MAT****a*** *ura3-1 leu2,3-112 his3-11 trp1-1 ade2-1 LYS2 can1-100 bar1∆ MIF2-3FLAG::KAN* |
| SBY8521 | *MAT****a*** *ura3-1::dsn1-S240A,S250A-12myc::URA3 leu2,3-112 his3-11 trp1-1 ade2-1 lys2∆ can1-100 bar1∆* |
| SBY8546 | *MAT****a*** *ura3-1 leu2,3-112 his3-11 trp1-1 ade2-1 LYS2 can1-100 bar1∆ MUB1-13myc::HIS3 MIF2-3FLAG::KAN* |
| SBY8550 | *MAT****a*** *ura3-1 leu2,3-112 his3-11 trp1-1 ade2-1 LYS2 can1-100 bar1∆ MUB1-13myc::HIS3 DSN1-HIS-FLAG::URA3* |
| SBY8551 | *MAT****a*** *ura3-1 leu2,3-112 his3-11 trp1-1 ade2-1 LYS2 can1-100 bar1∆ MUB1-13myc::HIS3 DSN1-HIS-FLAG::URA3 mif2-3* |
| SBY8552 | *MAT****a*** *ura3-1 leu2,3-112 his3-11 trp1-1 ade2-1 LYS2 can1-100 bar1∆ DSN1-HIS-FLAG::URA3 okp1-5::TRP1* |
| SBY8554 | *MAT****a*** *ura3-1 leu2,3-112 his3-11 trp1-1 ade2-1 LYS2 can1-100 bar1∆ DSN1-HIS-FLAG::URA3 cse4-323* |
| SBY8565 | *MAT****a*** *ura3-1 leu2,3-112 his3-11 trp1-1 ade2-1 LYS2 can1-100 bar1∆ MUB1-3FLAG::KAN* |
| SBY8572 | *MAT****a*** *ura3-1 leu2,3-112 his3-11 trp1-1 ade2-1 LYS2 can1-100 bar1∆ MUB1-13myc::HIS3 MIF2-3FLAG::KAN ubr2∆HIS3* |
| SBY8590 | *MAT****a*** *ura3-1 leu2,3-112 his3-11 trp1-1 ade2-1 LYS2 can1-100 bar1∆ MUB1-13myc::HIS3* |
| SBY8605 | *MAT****a*** *ura3-1 leu2,3-112 his3-11 trp1-1 ade2-1 LYS2 can1-100 bar1∆ DSN1-HIS-FLAG::URA3 pGAL-GFP-UBR2::KAN* |
| SBY8613 | *MAT****a*** *ura3-1 leu2,3-112 his3-11 trp1-1 ade2-1 lys2∆ can1-100 bar1∆ mub1∆HIS3 ndc10-1* |
| SBY8615 | *MAT****a*** *ura3-1::dsn1-S240A,S250A-3FLAG::URA3 leu2,3-112 his3-11 trp1-1 ade2-1 lys2∆ can1-100 bar1∆* |
| SBY8670 | *MAT****a*** *ura3-1 leu2,3-112 his3-11 trp1-1 ade2-1 LYS2 can1-100 bar1∆ MTW1-3GFP::HIS3 ndc80-1 mub1ΔHIS3* |
| SBY8703 | *MAT****a*** *ura3-1::dsn1-S240A,S250A-3FLAG::URA3 leu2,3-112 his3-11 trp1-1 ade2-1 lys2∆ can1-100 bar1∆ ubr2∆HIS3 cim3-1* |
| SBY8704 | *MAT****a*** *ura3-1::dsn1-S240A,S250A-3FLAG::URA3 leu2,3-112 his3-11 trp1-1 ade2-1 lys2∆ can1-100 bar1∆ cim3-1* |
| SBY8705 | *MAT****a*** *ura3-1 leu2,3-112 his3-11 trp1-1 ade2-1 LYS2 can1-100 bar1∆ DSN1-3FLAG::KAN ubr2∆HIS3 cim3-1* |
| SBY8706  SBY8766 | *MAT****a*** *ura3-1 leu2,3-112 his3-11 trp1-1 ade2-1 lys2∆ can1-100 bar1∆ DSN1-3FLAG::KAN cim3-1*  *MAT****a*** *ura3-1::DSN1-12myc::URA3 leu2,3-112 his3-11 trp1-1 ade2-1 can1-100 bar1∆ lys2∆* |
| SBY8773 | *MAT****a*** *ura3-1 leu2,3-112 his3-11 trp1-1 ade2-1 lys2∆ can1-100 bar1∆ ubr2∆HIS3 ndc10-1* |
| SBY8842 | *MAT****a*** *ura3-1::dsn1-S240A,S250A-12myc::URA3 leu2,3-112 his3-11 trp1-1 ade2-1 lys2∆ can1-100 bar1∆ pGAL-3HA-DSN1::KAN mub1∆NAT ubr2∆HIS3* |
| SBY8844 | *MAT****a*** *ura3-1::dsn1-S240A,S250A-12myc::URA3 leu2,3-112 his3-11 trp1-1 ade2-1 lys2∆ can1-100 bar1∆ pGAL-3HA-DSN1::KAN mub1∆NAT* |
| SBY8901 | *MAT****a*** *ura3-1::dsn1-S240A,S250A-12myc::URA3 leu2,3-112 his3-11 trp1-1 ade2-1 lys2∆ can1-100 bar1∆ pGAL-3HA-DSN1::KAN mub1∆NAT ubr2∆HIS3 rpn4∆HIS3* |
| SBY8911 | *MAT****a*** *ura3-1 leu2,3-112 his3-11 trp1-1 ade2-1 lys2∆ can1-100 bar1∆ MIF2-3FLAG::KAN mub1∆NAT* |
| SBY8944  SBY9428  SBY10959  SBY10960 | *MAT****a*** *ura3-1 leu2,3-112 his3-11 trp1-1::256lacO::TRP1 ade2-1 can1-100 LYS2 bar1Δ DSN1-6HIS-3FLAG::URA3 dad1-1::KAN*  *MAT****a*** *ura3-1 leu2,3-112 his3-11 trp1-1 ade2-1 lys2∆ can1-100 bar1-1*  *DSN1-13MYC::KAN mub1::HIS3 ipl1-321*  *MAT****a*** *ura3-1 leu2,3-112 his3-11 trp1-1 ade2-1 LYS2 can1-100 bar1-1*  *DSN1-13MYC::KAN mub1::HIS3*  *MAT****a*** *ura3-1::DSN1-12myc::URA3 leu2,3-112 his3-11 trp1-1 ade2-1 LYS2 can1-100 bar1-1 ubr2::HIS3* |
