# Supplementary material for: The Mub1/Ubr2 Ubiquitin Ligase Complex Regulates the Conserved Dsn1 Kinetochore Protein
Source: PLoS Genet. 2013 Feb 7;9(2):e1003216. doi: 10.1371/journal.pgen.1003216 (PMC3567142; doi:10.1371/journal.pgen.1003216)
Supplement: Table S4 — Plasmids used in this study. (DOCX) [file pgen.1003216.s006.docx]

**Table S4**. Plasmids used in this study.

| Plasmid | Description |
| --- | --- |
| pAG25 | *NATMX4* [[1](#_ENREF_1)] |
| pRS303 | *HIS3* (integrating) [[2](#_ENREF_2)] |
| pRS305 | *LEU2* (integrating) [[2](#_ENREF_2)] |
| pRS425 | *2 micron, LEU2* [[2](#_ENREF_2)] |
| pSB624 | *DSN1, CEN, URA3* [[3](#_ENREF_3)] |
| pSB737 | *pCMV-LacI-3FLAG, URA3* (integrating) [[4](#_ENREF_4)] |
| pSB812 | *3FLAG, KAN* [[5](#_ENREF_5)] |
| pSB818 | *MTW1-3GFP, HIS3* (integrating) [[6](#_ENREF_6)] |
| pSB944 | *dsn1-S264A, HIS3* (integrating) |
| pSB963 | *WT CEN3, 8LacO, TRP1* [[7](#_ENREF_7)] |
| pSB1097 | *DSN1, HIS3* (integrating) |
| pSB1099 | *dsn1-S240A,S250A, HIS3* (integrating) |
| pSB1104 | *dsn1-S240D,S250D, HIS3* (integrating) |
| pSB1108 | *dsn1-S240A,S250A,S264A, HIS3* (integrating) |
| pSB1110 | *DSN1-12myc, URA3 (integrating)* [[8](#_ENREF_8)] |
| pSB1111 | *dsn1-S240A,S250A-12myc, URA3* (integrating) |
| pSB1112 | *dsn1-S240D,S250D-12myc, URA3* (integrating) |
| pSB1113 | *DSN1-3FLAG, URA3* (integration) [[8](#_ENREF_8)] |
| pSB1114 | *dsn1-S240A,S250A-3FLAG, URA3* (integrating) |
| pSB1115 | *dsn1-S240D,S250D-3FLAG, URA3* (integrating) |
| pSB1122 | *DSN1-3GFP, HIS3* (integrating) |
| pSB1124 | *dsn1-S240D,S250D-3GFP, HIS3* (integrating) |
| pSB1138 | *dsn1-S240A,S250A,S264A-3GFP, HIS3* (integrating) |
| pSB1142 | *dsn1-S240A,S250A,S264A-3FLAG, URA3* (integrating) |
| pSB1312 | *dsn1-S240A,S250A,S264A-12myc, URA3* (integrating) |
| pSB1322 | *DSN1-12myc, 2 micron, LEU2* |
| pSB1323 | *dsn1-S240A,S250A-12myc, 2 micron, LEU2* |
| pSB1324 | *dsn1-S240D,S250D-12myc, 2 micron, LEU2* |
| pSB1326 | *pCMV-LacI-3FLAG, LEU2* (integrating) |
| pSB1540 | *dsn1-S240A,S250A,S264A-12myc, 2 micron, LEU2* |
| pSB1554 | *dsn1-S264A-3FLAG, URA3* (integrating) |
| pSB1590 | *DSN1-HIS-FLAG, URA3* (integrating) [[8](#_ENREF_8)] |
