# Supplementary material for: The Mub1/Ubr2 Ubiquitin Ligase Complex Regulates the Conserved Dsn1 Kinetochore Protein
Source: PLoS Genet. 2013 Feb 7;9(2):e1003216. doi: 10.1371/journal.pgen.1003216 (PMC3567142; doi:10.1371/journal.pgen.1003216)
Supplement: Table S5 — Oligonucleotides used in this study. (DOCX) [file pgen.1003216.s007.docx]

**Table S3**. Oligonucleotides used in this study.

| Purpose | Primers (all are listed 5’ to 3’) |
| --- | --- |
| *MIF2-13myc* | Forward **SB376** (GAC AAC GAC AAA GAA TTA GAC AGT ACG TTT GAC ACT TTT GGG GGT CGA CGG ATC CCC GGG TT)  Reverse **SB377** (CAG TAC ATA GCA TGC ATA ATG AGA ATA TTC ACA TCA TAA TTA TCG ATG AAT TCG AGC TT) |
| *DSN1-3HA, DSN1-13myc* | Forward **SB527** (AAG GGA TTA AGT TTA TCT TTC AGT AAA AAA CTG GAT TTA GGT CGA CGG ATC CCC GGG TT)  Reverse **SB528** (GCA GAA GTA TCC GAT TTT TTT TTG ATT TTT TCT TTT ATT TCG ATG AAT TCG AGC TCG TT) |
| *dsn1∆*  *DSN1-3FLAG* | Forward **SB573** (ATT GTA CCA CAC ACA AAT TTT TCT TTA TTT AGG TAG AGG GGT CGA CGG ATC CCC GGG TT)  Reverse **SB528** (GCA GAA GTA TCC GAT TTT TTT TTG ATT TTT TCT TTT ATT TCG ATG AAT TCG AGC TCG TT)  Forward **SB1682***(*ATT AAT CCT CAA CAG CTG TTG AAG GGA TTA AGT TTA TCT TTC AGT AAA AAA CTG GAT TTA GGT CGA AGG GAA CAA AAG CTG GAG CT)  Reverse **SB1683** (CTG TGT AAT GTT ACA TAT GCA GAA GTA TCC GAT TTT TTT TTG ATT TTT TCT TTT ATT TCA CTA TAG GGC GAA TTG GGT) |
| *mub1∆* | Forward **SB3209** (AGC GTA TAG AAC GTA ATT TGA AAT ATT CAA AAC AAA GAA AAG GTA AAG AAG GTC GAC GGA TCC CCG GGT T)  Reverse **SB3210** (TTT CAT GAT CTT GTT AAA CCT AGG TCA TCG AAA TCC CCT TGT ACT TCG ATG AAT TCG AGC TCG TT) |
| *ubr2∆* | Forward **SB2322** (TTA CTC GTC TCT GAT AGT AAA GGT AAG ATT CGT TAA CTA AAT TAA TAG CTA CTT AAC AAG CAC GCC GGA TCC CCG GGT TAA TTA A)  Reverse **SB2323** (TCT TTT GAT TTT TCG GAT ACG CTA ATT TCG TAG CAA TTT TGA ATG ACT AGA CAT TTG TTG GAT AAG AAT TCG AGC TCG TTA AAC) |
| *pGAL-3HA-DSN1* | Forward **SB481** (GAT AAA AGC ACA AAG TAC CAG GCA GAG TTG TGA AAC CGG GAA TTC GAG CTC GTT TAA AC**)**  Reverse **SB482** (CGG CGG CGT ACC GGA GAC CGT TTG TGT GGG TTC CAG ACT GCA CTG AGC AGC GTA ATC TG) |
| *rpn4∆* | Forward **SB2171** (TTG TAT CTT TTC AAA AGT TTT CTA GAA TTT TCA AGC AAT CCG GAT CCC CGG GTT AAT TAA)  Reverse **SB2170** (GGT TTT CTT CTT TTA TCT CCT ATA TAA TTT GTA ACC TTA AGA ATT CGA GCT CGT TTA AAC) |
| *MIF2-3FLAG* | Forward **SB2489** (TCA GAA GAC GCT AAC GAT GAC AAC GAC AAA GAA TTA GAC AGT ACG TTT GAC ACT TTT GGG AGG GAA CAA AAG CTG GAG CT)  Reverse **SB2490** (ATG GAC CTA GTT ATA TTT CTT CAG TAC ATA GCA TGC ATA ATG AGA ATA TTC ACA TCA TAA CTA TAG GGC GAA TTG GGT) |
| *MUB1-3FLAG* | Forward **SB2511** (GTT CAC TCC TAA TAT ATC CAC CAC CGT ACC TGA TGA AAT AAG CAA TAG GGA TGA AAA TAG CAT ACC TGA GAG GGA ACA AAA GCT GGA GCT)  Reverse **SB2512** (AAA CAA AAA ACT CCG TTA ATG AAT ATT TCA TGA TCT TGT TAA ACC TAG GTC ATC GAA ATC CCC TTG TAC TCT ATA GGG CGA ATT GGG T) |
| *MUB1-13myc* | Forward **SB2086** (AAT ATA TCC ACC ACC GTA CCT GAT GAA ATA AGC AAT AGG GAT GAA AAT AGC ATA CCT GAG GGT CGA CGG ATC CCC GGG TT)  Reverse **SB2087** (ACT CCG TTA ATG AAT ATT TCA TGA TCT TGT TAA ACC TAG GTC ATC GAA ATC CCC TTG TAC TCG ATG AAT TCG AGC TCG TT) |
| *dsn1-S240A, S250A* | Forward **SB1627** (AAG GAA AAA AgC AAT CGG AAG TCA GAG AGG TAG AAG ATT GgC TAT GTT AGC)  Reverse **SB1628** (GCT AAC ATA GcC AAT CTT CTA CCT CTC TGA CTT CCG ATT GcT TTT TTC CTT) |
| *dsn1-S240D, S250D* | Forward **SB1680** (GGA AAG GAA AAA Ag**a c**AT CGG AAG TCA GAG AGG TAG AAG ATT GG**a** TAT GTT AGC ATC)  Reverse **SB1681** (GAT GCT AAC ATA tCC AAT CTT CTA CCT CTC TGA CTT CCG ATg tCT TTT TTC CTT TCC) |
